# Evidence of Neurotoxicity of Ecstasy: Sustained Effects on Electroencephalographic Activity in Polydrug Users

**DOI:** 10.1371/journal.pone.0014097

**Published:** 2010-11-23

**Authors:** Michael Adamaszek, Alexander V. Khaw, Ulrike Buck, Burghard Andresen, Rainer Thomasius

**Affiliations:** 1 Neurologisches Rehabilitationszentrum Leipzig, University of Leipzig, Bennewitz, Germany; 2 Clinic of Psychiatry and Psychotherapy, University Medical Center Hamburg-Eppendorf, Hamburg, Germany; 3 Department of Neurology, University of Greifswald, Greifswald, Germany; 4 Department of Psychiatry, Hospital Rissen, Hamburg, Germany; 5 Deutsches Zentrum für Suchtfragen des Kindes- und Jugendalter, University Medical Center Hamburg-Eppendorf, Hamburg, Germany; University of Granada, Spain

## Abstract

**Objective:**

According to previous EEG reports of indicative disturbances in Alpha and Beta activities, a systematic search for distinct EEG abnormalities in a broader population of Ecstasy users may especially corroborate the presumed specific neurotoxicity of Ecstasy in humans.

**Methods:**

105 poly-drug consumers with former Ecstasy use and 41 persons with comparable drug history without Ecstasy use, and 11 drug naives were investigated for EEG features. Conventional EEG derivations of 19 electrodes according to the 10-20-system were conducted. Besides standard EEG bands, quantitative EEG analyses of 1-Hz-subdivided power ranges of Alpha, Theta and Beta bands have been considered.

**Results:**

Ecstasy users with medium and high cumulative Ecstasy doses revealed an increase in Theta and lower Alpha activities, significant increases in Beta activities, and a reduction of background activity. Ecstasy users with low cumulative Ecstasy doses showed a significant Alpha activity at 11 Hz. Interestingly, the spectral power of low frequencies in medium and high Ecstasy users was already significantly increased in the early phase of EEG recording. Statistical analyses suggested the main effect of Ecstasy to EEG results.

**Conclusions:**

Our data from a major sample of Ecstasy users support previous data revealing alterations of EEG frequency spectrum due rather to neurotoxic effects of Ecstasy on serotonergic systems in more detail. Accordingly, our data may be in line with the observation of attentional and memory impairments in Ecstasy users with moderate to high misuse. Despite the methodological problem of polydrug use also in our approach, our EEG results may be indicative of the neuropathophysiological background of the reported memory and attentional deficits in Ecstasy abusers. Overall, our findings may suggest the usefulness of EEG in diagnostic approaches in assessing neurotoxic sequela of this common drug abuse.

## Introduction

Since the late 1980s, Ecstasy has been especially known in the so-called “techno”-scene as a recreational drug due to its specific psychotropic effects, characterized in psychopharmacologic terms as an entactogen. However, numerous hazards related to this drug and its substantial compounds as 3,4-Methylenedioxymethamphetamine (MDMA) have been disclosed. Besides various medical and diverse psychiatric disturbances, there is striking evidence for cognitive impairments such as memory and attention associated with Ecstasy use [1]–[5].

In regard to research findings in animal models, MDMA as the principal compound of Ecstasy revealed neurotoxic effects predominantly in serotonergic structures of the central nervous systems (CNS) with no or incomplete regeneration in neocortical as well as other distinct brain structures like the limbic system [6]–[9]. More precisely, neuroimaging approaches in humans like positrone emission tomography (PET) and functional MRI, or cerebrospinal fluid (CSF) analysis support clear evidence of specific neurotoxicity effects of Ecstasy consumers in the serotonergic system [2], [10]. More interestingly for our approach, EEG data from subjects with poly-drug abuse including recent Ecstasy use showed disturbances in brain function with altered activities in the Alpha and lower Beta band, but, moreover, a reduced interhemisperic EEG coherence [11]. Several reports of EEG analyses and brainstem acoustic evoked potentials (BAEP) mainly pointing to neuropathophysiological changes among Ecstasy users, indicating a selective neurotoxicity within the serotonergic system of the CNS [12]–[15]. Among the numerous serotonergic and noradrenergic neurotransmitter systems, primarily 5-HT-specific projections from the raphe nuclei to thalamic, hypothalamic and hippocampal areas, and furthermore to the visual, frontal and temporal visual association cortices, are considered a central potential target [16], [17]. 5-hydroxytryptamin is mainly synthesized in the raphe nuclei and modulates as a critical neurotransmitter for different functions like wake-sleep-rhythm, behavioural arousal, and attention [17]. Thus, disturbances of these functions due to selective neuropathogeneity of Ecstasy may be expected. Although numerous clinical reports support the neuroanatomical background for Ecstasy neurotoxicity in humans, published data are still incomplete and controversial, partly because of methodological restrictions [18].

According to the still prominent and robust neurophysiologic findings in Ecstasy users, the aim of the present study was to detect whether EEG activity is altered in an extended representative sample of former Ecstasy users. The present study, as part of a great investigation for registering pathological features of Ecstasy consumption, intends to enlighten the discussion whether disturbances of serotonergic pathways due to neurotoxic effects of the principal components of Ecstasy commonly distributed within European areas are disclosable in neuroimaging techniques such as the EEG. If so, the EEG comfortable for neurophysiological requests everywhere may be recommendable at least in diagnostic approaches to calculate neurotoxicologic effects of Ecstasy in suspected humans.

## Materials and Methods

### Subjects

The study was conducted for investigation on permanent sequelae of Ecstasy use in subjects associated with the “techno-scene”. This investigation aimed to identify psychological and physical health risks of Ecstacy and to develop a risk-classification scheme for certain subgroups of polydrug users. In regard to a mutidisciplinary study concept, the present study focused the investigation of possible alterations of certain EEG variables according to Ecstasy misuse.

One-hundred and fifty-seven men and women were enrolled in this study. One-hundred and five subjects had ingested variable quantities of Ecstasy in addition to the use of “typical drugs” like amphetamines, hallucinogenes, cannabinoids and cocaine in various combinations. Forty-one subjects served as controls for the Ecstasy users, i.e. they had similar patterns of polydrug use, but had never ingested Ecstasy. A second control group of eleven subjects had never ingested any drugs, termed drug naives. For estimation of dose-effect-relationships, the Ecstacy user group was divided into three subgroups according to the cumulative total amount of Ecstacy tablet ingestion: 1–99 tablets defined “tasting users”, 100–499 tablets defined “occasional users”, and 500 or more “permanent users”, respectively. Substantial inclusion criteria was the relationship to the “techno scene”. Subjects were recruited mainly by inquiries in well known locations of the “techno”-scene of Hamburg, Germany. All enrolled subjects were examined clinically and checked for internal, neurologic and psychiatric disorders as exclusion criteria.

Laboratory analyses of hair samples were performed to validate the self-reported drug history. Details on drug history and toxicologic laboratory investigations are already published elsewhere [19], [20]. All participants were informed about design and background of the study, and gave written consent, as approved by the local ethics committee of the Medical Facility at the University of Hamburg, Germany. This procedure was constituted and considered without any exceptions in regard to the Declaration of Helsinki.

### Procedures

Ten minutes of resting EEG were employed for statistical evaluation. Two minutes with photostimulation and three minutes with hyperventilation were also recorded for clinical assessment of lowered seizure threshold. 19 electrodes recording in a configuration according to the standard 10-20-system was applied. Analogue measurements of EEG signals were performed with a time constant of 10 s, and the sampling rate was 256 per second.

For analysis of EEG power spectra, artefact-free sequences of at least two seconds were used. The power spectra were restricted to the standard band ranges in electroencephalography: Delta: 0.5–3.5 Hz, Theta: 3.5–7.5 Hz, Alpha: 7.5–13.5 Hz und Beta: 13.5–30.0 Hz. Special variables were formed for a closer differentiation of possible shifts to slower or faster activities: Theta 1: 3.5–5.0 Hz, Theta 2: 5.0–7.5 Hz, Alpha 1: 7.5–9.0 Hz, Alpha 2: 9.0–11.0 Hz, Alpha 3: 11.0–13.5 Hz; Beta 1: 13.5–20.0 Hz, Beta 2: 20.0–22.0 Hz; Beta 3: 22.0–30.0 Hz. Power computation was drawn from occipital (O1, O2) and parasagittal (F3, F4; C3, C4; P3, P4) channels. Spectral power in the Alpha band was further analyzed at 0.5, and Theta ranges at 1.0 Hz intervals. Finally, lower Alpha- and sub-Alpha-power during the first two minutes of EEG acquisition were compared. Delta activity was only assessed from qualitative EEG because of the high amount of artifacts.

EEG recordings and analyses of power spectra were conducted with the Neurofile system by Nihon Kohden (V2.91; Japan). Calculation of digital data from the 2-sec-epochs were conducted with a digitalizing rate of 256 per second.

### Statistical analysis

Analysis of Variance (ANOVA) was applied in calculating the differences of the defined EEG bands in quantitative EEG, thus analysing each EEG data for all groups of Ecstasy and non-Ecstasy usage. Furthermore, Analysis of Covariance (ANCOVA) was employed for the effects of concomitant drugs. Associations were estimated by Pearson's correlation. Differences between groups were assessed by the post hoc Scheffé-test (90% resp. 95% confidence interval for two-way testing). P-values ≤0.05 and ≤0.01 were considered as significant and highly significant, respectively.

## Results

157 female and male subjects with a mean age of 22 years (+3.70) were enrolled in this study. 101 subjects had a history of Ecstasy consumption, and the median time range of abstinence from Ecstasy was around 5 months (3 days minimal, 8 years maximal). 9 users with a total ingestion of less than 100 Ecstasy tablets were abstinent on an average of 9.8 months, 56 users with 100 up to 499 tablets on an average of 3.0 months, and 36 users with more than 500 tablets on an average of 3.4 months, respectively. 41 subjects with a comparable polysubstance use of common drugs like cannabis, cocaine and amphetamines, but without Ecstasy, represent drug controls. Furthermore, 11 subjects had not had any experiences with illicit drugs, thus representing drug naives (see table 1). Subjects with a medium and severe Ecstasy ingestion behaviour yielded a higher ingestion rate of other illicit drugs in subjects. Control subjects with a polysubstance use were quite comparable in their consumption behaviour of cannabis ingestion, whereas remaining drugs were less frequently represented in this control group. Noteworthy in regard to the assessments of each participant, toxicologic analyses of hair samples revealed an agreement of 91.3% to self-reported drug history.

**Table 1:** Prevalences of drug misusers and their consumptions.

*Psychotropic substances*	*Polysubstance use with Ecstasy consume*												*Controls*					
	*all* *N = 101*			*tasting users* *N = 9*			*occasional users* *N = 56*			*permanent users* *N = 36*			*polysub-stance use without Ecstasy consume* *N = 41*			*drug naives* *N = 11*		
***prevalences***	30	6	12	30	6	12	30	6	12	30	6	12	30	6	12	30	6	12
*Ecstasy*	56	74	84	22	56	56	64	79	88	53	72	89	-	-	-	-	-	-
*Alcohol*	78	87	87	100	100	100	71	80	80	83	94	94	94	94	94	64	93	93
*Cannabis*	59	70	75	44	67	89	61	75	77	61	61	69	75	86	86	-	-	7
*Amphetamines*	28	52	62	-	11	22	27	54	63	36	61	72	3	8	11	-	-	-
*Cocaine*	34	61	67	11	44	44	36	59	70	36	69	69	11	22	22	-	-	-
*Halluzinogenes*	16	33	50	11	11	22	18	38	50	14	31	56	3	6	11	-	-	-
*Heroine*	2	2	3	-	-	-	4	4	5	-	-	-	-	-	-	-	-	-
*other Opiates* *or Analgesics*	-	-	1	-	-	-	-	-	2	-	-	-	-	-	-	-	-	-
*Sedatives*	4	6	6	-	11	11	5	5	5	3	6	6	-	-	-	-	-	-
*Sniffle substances*	2	8	12	-	-	-	4	7	11	-	11	17	3	3	3	-	-	-
*Other drugs*	7	16	24	-	-	-	11	20	27	3	14	25	-	-	8	-	-	-

30-day-prevalence (30), 6-months-prevalence (6) and 12-months-prevalence (12) of drug consumption regarding differences in group and consumption order (results in percentage).

Comparing the conventional EEG activity bands of Ecstasy users with controls, high Ecstasy users showed a significant increase of power for Beta bands (F 3.41; p = 0.029). Moreover, medium and high Ecstasy users yielded an augmentation of slow frequencies in the Theta range (nonsignificant). Low Ecstasy users revealed a trend to the faster Alpha subband, whereas high Ecstasy users showed a trend to lower Alpha subband.

**Figure 1:**
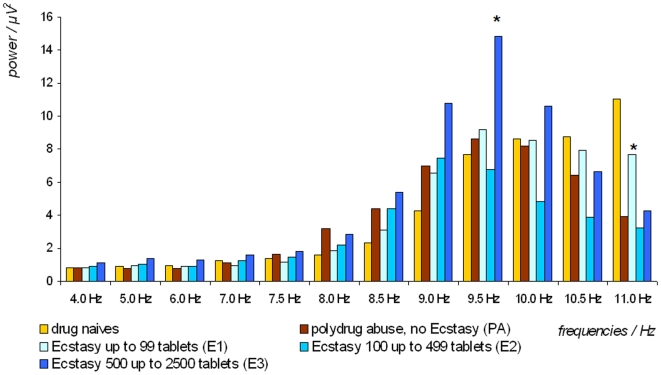
EEG subbands of Alpha and Theta activities in regard to drug consumption order. Histogram of spectral power by electroencephalographic frequency in 1.0 Hz steps within the Theta-band, and in 0.5 Hz steps within the Alpha-band, according to groups of polydrug-users with and without Ecstasy consumption and drug naives, in a study cohort of 146 polydrug-users and 11 drug naives as controls.

Comparing all groups at the analysis of Alpha band power subdivided by 0.5 Hz steps, a dominant frequency at 9 Hz among medium Ecstasy users was detected (see figure 1). High Ecstasy users and poly-drug users with no history of Ecstasy showed a peak at 9.5 Hz, while low Ecstasy users showed a peak of dominant frequency at 11 Hz (F 3.87; p = .001).

**Figure 2:**
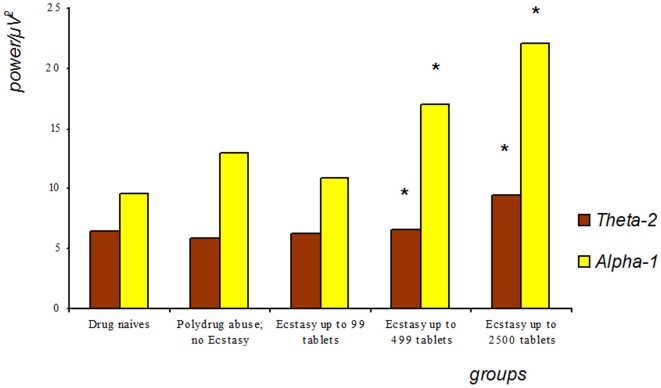
Comparison of lower Alpha and upper Theta activities of the first two minutes of recording. Spectral power of upper Theta-band (theta-2, i.e. 5.0–7.5 Hz) compared with lower Alpha-band (alpha-1, i.e. 7.5–9.0 Hz) by groups of polydrug-users with and without Ecstasy consumption and drug naives (abbreviations coded as in figure 1) during the first 2 minutes of EEG registration.

Analysing the slow activities in the first two minutes of EEG recording, medium and high Ecstasy users yielded increased power for lower Alpha (F 2.98; p = .047) and upper Theta ranges (F 3.01; p = 0.014) (see figure 2).

We found a positive correlation between categorized Ecstasy consumption and an increase of 5 Hz and, within the first two minutes of EEG recording, the low Alpha and upper Theta subband spectral power (see table 2).

**Table 2:** Overview of correlative effects of consumed drugs and EEG variables.

*Variables of EEG//Drugs*	*5.0* *Hz*	*9.0* *Hz*	*9.5* *Hz*	*Theta-2/2 minutes*	*Alpha-1/2 minutes*
*Ecstasy*	0.240(.002)	0.144(.071)	0.152(.059)	0.204(.010)	0.231(.004)
*Cannabinoides*	−0.048(.562)	−0.070(.398)	−0.037(.657)	−0.085(.305)	−0.064(.438)
*Hallucinogenes*	−0.029(.792)	−0.076(.491)	−0.121(.273)	0.003(.980)	0.008(.944)
*Amphetamines*	−0.037(.712)	−0.058(.565)	−0.083(.409)	−0.070(.482)	−0.035(.727)
*Cocaine*	−0.013(.897)	−0.056(.575)	−0.50(.621)	−0.021(.838)	0.052(.606)

Correlation coefficients for selected spectral bands from quantitative EEG by drug categories in a sample of 105 polydrug-users including Ecstasy use (Pearson's correlation, p-value in parentheses); 5.0; 9.0; and 9.5 Hz indicates spectral power at discrete EEG frequency band; Theta-2 and Alpha-1 of first 2 minutes indicates spectral power of 5.0 up to 7.5 Hz for Theta-2, and 7.5 up to 9.0 Hz for Alpha-1.

Analysis of covariates (ANCOVA) found a significant main impact of Ecstasy and no relevant impact of other concurrent drugs (Amphetamines, Cannabinoides, Hallucinogenes, Cocaine) on the above mentioned changes in spectral power (F-value for Ecstasy at 5 Hz 5.48; p = .006, at 9.5 Hz F = 3.51; p = .034, at 11.0 Hz F = 6.06; p = .003). During the first two minutes of EEG recording, the effect of Ecstasy on the upper Theta subband power approximated statistical significance (F 2.95; p = .057).

## Discussion

Principal findings of our study were an increase in absolute power of Beta, low Alpha and Theta activities in association with a marked decrease in the frequency of dominant activity in Ecstasy users with moderate to high life-time dosages. More particularly, the observation of increases of low Alpha and Theta activities was already pronounced at the early recording session. A dose-dependent increase was found around 5 and 20 Hz in subjects with a medium to high Ecstasy use. Another interesting finding was a strong power of Alpha activity at 11 Hz in Ecstasy users with low life-time dosages, assuming a normal EEG. Any influences of other sedative or stimulating drugs such as cannabis, amphetamines or hallucinogenes may be ruled out by ANCOVA. Furthermore, no other typical EEG patterns suggesting toxic effects on the CNS, such as generalized slowing, rhythmic delta activity or triphasic complexes, were observed. These results are in line with former EEG studies using similar designs [11], [12], [14].

The results may support the assumption of a specific neurotoxicity of Ecstasy and its frequent compound MDMA to serotonergic neurotransmission systems in human CNS. In addition to noradrenergic and dopaminergic neuronal circuits between brainstem and midbrain structures, like the locus coeruleus, the median forebrain bundle and its bidirectional connections to posterior and forebrain areas, serotonergic neurotransmission is of special interest in regard to the sleep-wake-rhythm and vigilance regulation [17], [21]. Indeed, disturbed serotonergic neurotransmission may result in increases of Theta and low Alpha activities in EEG [17]. The assumption that Ecstasy contributes substantially to our EEG findings may be additionally supported by data on selective serotonin reuptake inhibitors like fluoxetine, showing a close relationship between the activities of serotonergic transmitting systems and changes in Alpha and Beta spectra, accompanied with clinical states of awakeness [22], [23]. Specific serotonergic projections of the dorsal and median raphe nucleus to hypothalamic, frontal and occipital areas are affected by neurotoxic agents like Ecstasy, and, therefore, are implemented in modulating attention, memory and executive tasks [24], [25]. Therefore, a linking of the neurobiologic and neurophysiologic approach appears more reasonable [4], [26]. Thus, the clinical impact of these well reported altered EEG activities and our findings have to be considered with special interest. Clinical EEG research underscores the crucial relevance of vigilance regulation networks for high order cognitive and affective functions [21], [27]. A more recent study did indeed hallmark a strong impact of observed vigilance dynamics in EEG to fMRI signals, which are quite in agreement for certain cognition procedures and its topographic brain areas, in particular the frontal and temporal cortices [28]. However, although McKenna recognized vigilance disturbances in EEG recordings among Ecstasy users [29], specific analyses have not been performed so far. This neglect of analysing EEG data more precisely on this topic may be due to the particular consideration of results obtained with newer neuroimaging techniques such as cerebral PET or MRI and its previous elucidative positive correlations to cognitive and also to emotional dysfunctions in humans with a long-term abuse of Ecstasy [2], [20].

Thomasius in his first major search for neurotoxic sequela in one-hundred and five long time Ecstasy users, of which our EEG recordings were obtained, found several neuropsychiatric sequela, which have been already published elsewhere [19]. Interestingly, subjects with a medium and high Ecstasy use showed impairments in short term and working memory, confirming previous results of cross-sectional as well as longitudinal studies of cognitive impairment in Ecstasy users in different neuropsychological and imaging approachments [1], [4], [30]. Although we did not compare our EEG data with the obtained neuropsychologic data of Thomasius' approach in more detail due to editorial restrictions, our EEG findings may correspond to these particular memory disturbances due to Ecstasy misuse [31]–[33]. In this line, the increased power in Theta band in medium and high Ecstasy users may indicate functional alterations in hypothalamus or hippocampus, though parahippocampal and the medial frontal and posterior regions could be shown as highly correlated to subsequent memory-dependent Theta power in EEG [24], [34], [35]. This assumption is supported by learning and memory dysfunctions and aberrant regeneration in monkeys exposed to Ecstasy compounds [8], [34]. In healthy humans, increase of low frequencies in EEG show a clear correlation with decline of sustained attention, which is necessary in preceding memory efforts [25].

Like most previous studies on persisting effects of Ecstasy in humans, our study is subject to the methodological problem of polydrug use. However, pure Ecstasy users are still rare; therefore, investigations of isolated Ecstasy effects have been unsuccessful and do not appear feasible [13], [18], [30]. By investigating a quite large sample of Ecstasy users, our analyses still reached a stronger statistical power compared to previous publications. Moreover, accounting for possible interactions with concurrent drugs ascertained the effect of Ecstasy on EEG spectral changes. Common recreational drugs like alcohol, psychotropic stimulants or cannabis usually do not yield EEG patterns as found in our study. In particular, these CNS active drugs such as cannabis with its well-recognized risk factor for neuropsychiatric and neuropsychologic disorders are quite removed from the pathophysiologic pathway of Ecstasy, as shown for cannabinoids with its neuroprotective actions and especially its blocking properties of MDMA-induced neurotoxicity in laboratory animals [27]. Nevertheless, one may argue that a specific influence of serotonin as well as disturbed serotonergic pathways due to selective toxic agents such as MDMA could not be detected in neurophysiologic procedures such as EEG. This objection seems to be unjustified, though our EEG findings are well in line with previous EEG investigations of human Ecstasy consumers in different fashions [11], [14]. The lower specificity of EEG in focusing selected neurotransmitter systems, especially the serotonergic, and their relationships to functional neuronal networks represents a common disadvantage in all neuroimaging techniques. We still favoured the EEG due to its flexibility in analysing the EEG power in more detail, as it is well accepted in neuroscientific research capturing features of brain disturbances in regard to toxicologic effects as noted for Ecstasy and its frequent compounds. Moreover, analysis of covariate influences of concomitant drugs to altered EEG power in our study yielded a main effect for Ecstasy as the principal contributing variable. We also did not conduct specific genetics analysis, in particular serotonin receptor or transporter mechanisms in identifying special polymorphisms, which may contribute to our study. Nevertheless, these very new aspects in studying serotonin pathways have also not been considered in other comparable study designs, but further studies implementing these exciting approaches in pathophysiologic Ecstasy neurotoxicity aspects are anxiously awaited.

Besides the clinical avenues such as neuropsychological inventories, further investigations with a longitudinal design proving lasting Ecstasy effects on EEG in polydrug users are of special interest and may be beneficial for the ongoing discussion of the neurotoxicity particularly of common substances of Ecstasy in humans [36]. Neurophysiologic approaches in investigating the neurotoxicity of Ecstasy in humans are of highly promising value, in particular linking the frequent observation of disturbed skills like working memory and attention.
